# Solid pseudopapillary neoplasm of the pancreas with hepatic metastases: problems and strategies

**DOI:** 10.3389/fonc.2024.1410888

**Published:** 2024-07-19

**Authors:** Xiaocheng Li, Jiaxin Ren, Jianji Ke, Peng Jiang, Liang Guo, Li Zhang, Wei Han, Yahui Liu, Bai Ji

**Affiliations:** ^1^ Department of Hepatobiliary and Pancreatic Surgery, General Surgery Center, the First Hospital of Jilin University, Changchun, China; ^2^ Department of Neurology, the First Hospital of Jilin University, Changchun, China; ^3^ Department of Pathology, the First Hospital of Jilin University, Changchun, China; ^4^ Department of Radiology, the First Hospital of Jilin University, Changchun, China

**Keywords:** solid pseudopapillary neoplasm, hepatic metastases, diagnosis, treatment, prognosis

## Abstract

**Background:**

Solid pseudopapillary neoplasms of the pancreas with hepatic metastases are infrequent and difficult to diagnose, and treatment is uncertain.

**Methods:**

A retrospective analysis of clinical data from patients with pancreatic solid pseudopapillary neoplasm (SPN) hepatic metastases who underwent surgery at the First Hospital of Jilin University from January 2005 to December 2021 was conducted. A total of 287 patients with SPN were included in the study, of which 8 (3%) developed liver metastases, all of whom were treated surgically and recovered well after surgery. The clinical presentation, imaging features, surgical treatment, histopathological examination, and postoperative follow-up data (mean 70 months; range 28–138 months) of the patients were recorded and analyzed. Clinical response strategies can be derived by reviewing previous studies on hepatic metastases of SPNs.

**Results:**

For resectable hepatic metastases from pancreatic solid pseudopapillary neoplasms, early surgery with total resection of the primary tumor and metastasis has shown great efficiency and is associated with patient good prognosis. In patients presenting unresectable hepatic metastases, aggressive tumor reduction surgery resulted in the alleviation of clinical symptoms and reduction of tumor burden while potentially achieving long-term survival.

**Conclusion:**

For hepatic metastases of SPNs, a preoperative liver tissue biopsy is beneficial for a definitive diagnosis. Surgery demonstrates excellent therapeutic efficacy and is considered the preferred curative treatment approach. This paper presents clinical experiences with SPN-related hepatic metastases at the Affiliated Hospital of Jilin University, which can be used to guide patient counseling in clinical practice.

## 1 Introduction

The solid pseudopapillary neoplasm (SPN) was first reported by Frantz in 1959 (1). Historically, SPN has been defined using terms including pancreatic papillary epithelial tumors, solid and papillary tumors of the pancreas, and Hamoudi or Frantz tumors named after their discoverers (2). Until 1996, the World Health Organization (WHO) defined SPN as a junctional tumor of the pancreas, or an indeterminate potentially malignant tumor. It was only in 2010 that the WHO further defined it as a low-grade malignant tumor (1–3). Although SPN of the pancreas is usually non-invasive, around 10-15% of tumors are estimated to invade (4, 5). Extra-pancreatic SPN metastases might present in several tissues. However, these occur mostly in the liver and less commonly in the lymph nodes and peritoneum. Involvement of the portal vein, spleen, duodenum, omentum, colon, lungs, and blood vessels has also been reported (1). For resectable tumors, surgical removal with negative margins is the primary treatment, but unlike pancreatic cancer, overall two-year survival rates of 97% and five-year survival rates of 95-96% after complete resection can be achieved (1, 6). In contrast, treatment options for patients with unresectable tumors are limited and still debatable among the scientific community. To date, around 100 cases have been reported in the literature, mostly in single case reports. Moreover, several studies present incomplete clinical data. Here, we report eight cases of histologically confirmed liver metastases from solid pseudopapillary tumors of the pancreas that underwent surgical treatment at the First Hospital of Jilin University between 2005 and 2021. We reviewed the relevant literature and discussed SPN clinical features, imaging manifestations, differential diagnosis, treatment modalities and prognosis in a comprehensive manner.

## 2 Materials and methods

We retrospectively analyzed the data of patients with pancreatic SPN who underwent surgical treatment and received histological confirmation at the First Hospital of Jilin University from January 2005 to December 2021. During this period, a total of 287 SPN patients with complete data from our hospital, including data from 8 patients with hepatic metastases of pancreatic SPN, were included in this study. Clinical information was obtained by the attending hepatobiliary and pancreatic surgeon before and after surgery as well as during routine postoperative follow-up. Computed tomography (CT)/magnetic resonance imaging (MRI) and pathological observations were summarized by experienced professionals in the respective fields. The extent of surgical resection, categorized according to intraoperative conditions and postoperative imaging, included gross total resection (GTR, complete resection of the lesion) in seven cases (87.5%) and subtotal resection (STR, partial or incomplete resection) in one case (12.5%). All specimens underwent histopathological examination to ascertain the diagnosis. One case from the series has already been published as an image presentation (7). Postoperatively, all patients included in this study underwent routine clinical and radiological evaluation as well as postoperative follow-up. This study was approved by the Ethics Committee of the First Hospital of Jilin University, and informed consent was obtained from all patients. The study complied with The PROCESS 2018 statement (8). In addition to this, we reviewed the relevant literature and thoroughly discussed the clinical features, imaging manifestations, differential diagnosis, treatment modalities, and prognosis of patients with SPN.

## 3 Results

### 3.1 Patient demographics and clinical characteristics

**Figure 1:**
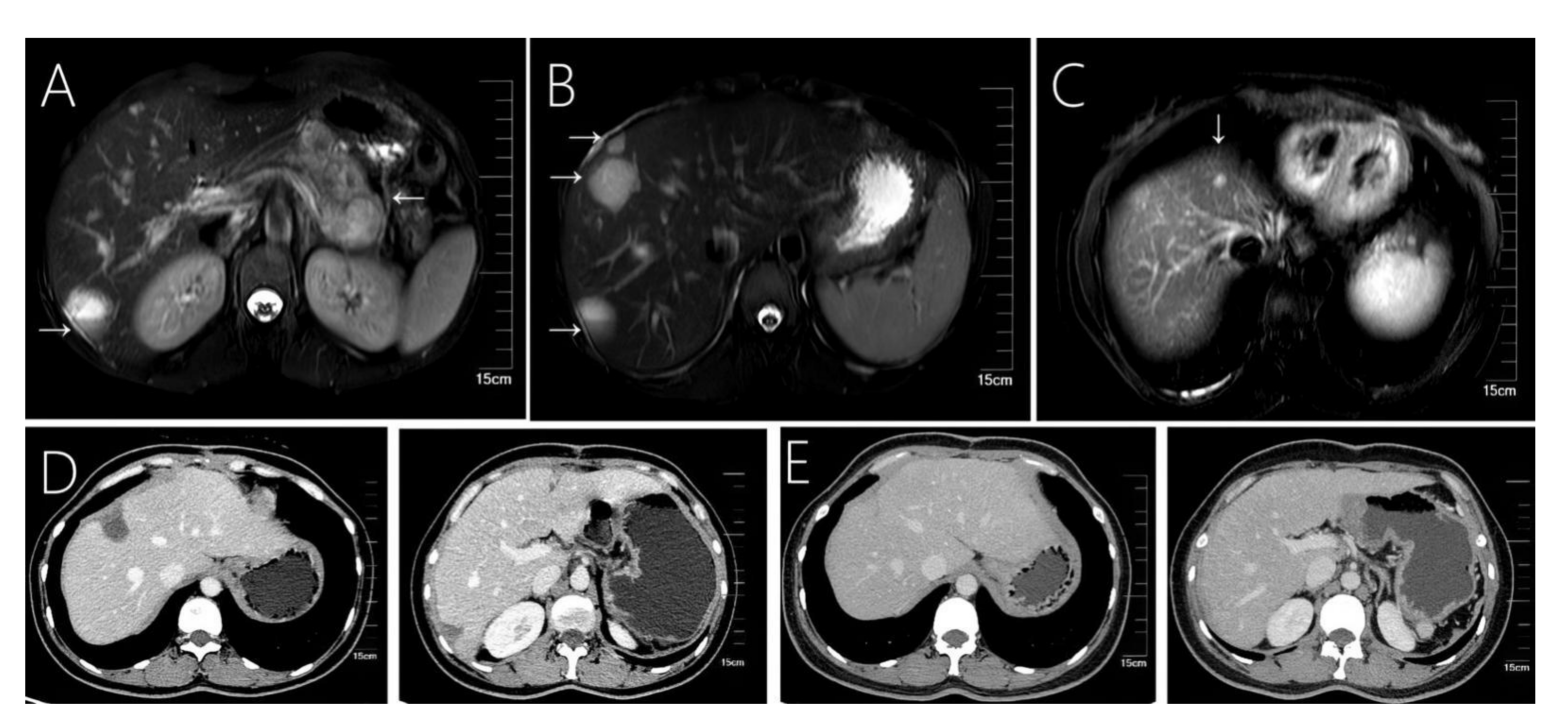
Case 1 shows that 31-year-old female with solid pseudopapillary neoplasm of the pancreas with hepatic metastases. **(A)** Fast spin-echo T2-weighted image shows a mixed cystic-solid structure within the mass. **(B, C)** A total of 4 cystic-solid mixed metastases are visible in the liver. **(D)** A small amount of effusion in the liver operated area was found at the 6th month of follow-up after surgery. **(E)** Images from follow-up at 36-months, showing a regular liver morphology and no metastatic lesions.

**Figure 2:**
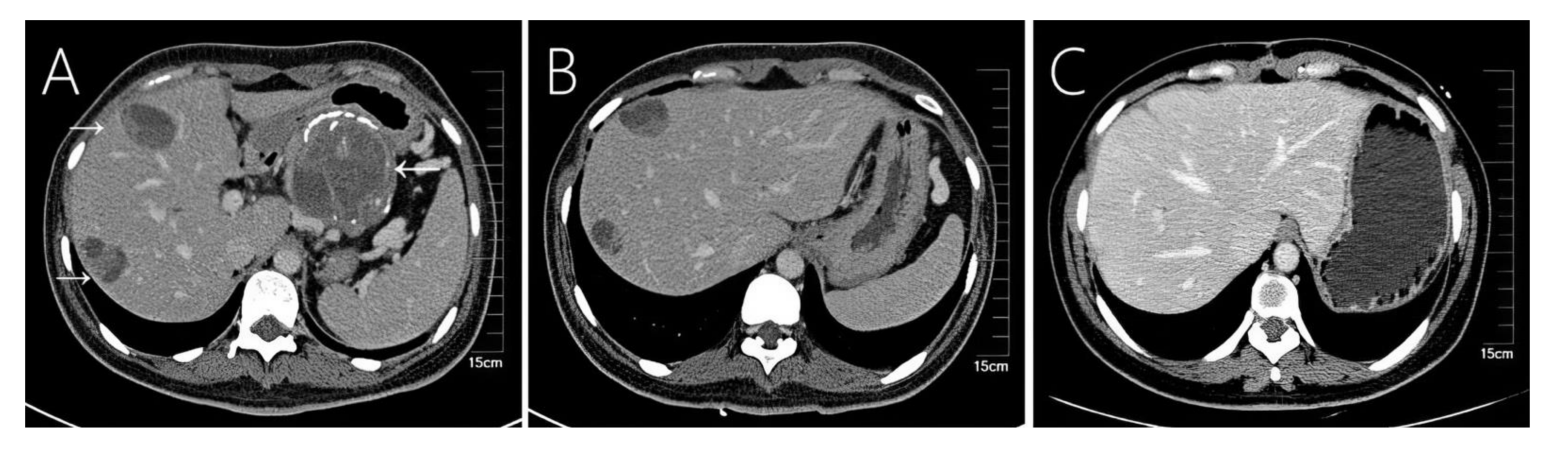
Case 2 shows a 36-year-old woman’s abdomen with contrast-enhanced CT scan showing a well-encapsulated heterogeneous mass in the tail of the pancreas. **(A)** The primary tumor showed eggshell-like calcification, and two liver metastases were seen in the liver, showing a mixed structure of cysts and solids. **(B)** Compared with preoperative CT at almost the same level, **(C)** the 54th month postoperative follow-up shows regular liver morphology and no other liver metastases.

**Figure 3:**
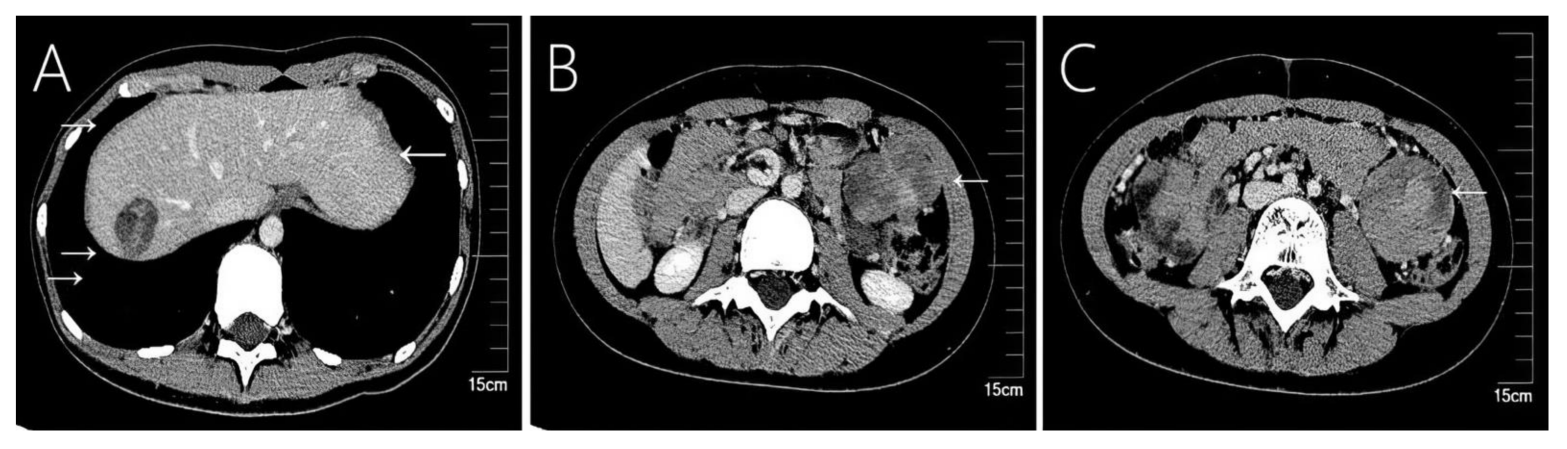
Case 6 shows a 9-year-old girl who underwent emergency surgery for spontaneous rupture of a solid-pseudopapillary neoplasm of the pancreas, and 78 months later tumor recurrence in the **(A)** liver, **(B)** omentum, and **(C)** colon.

**Figure 4:**
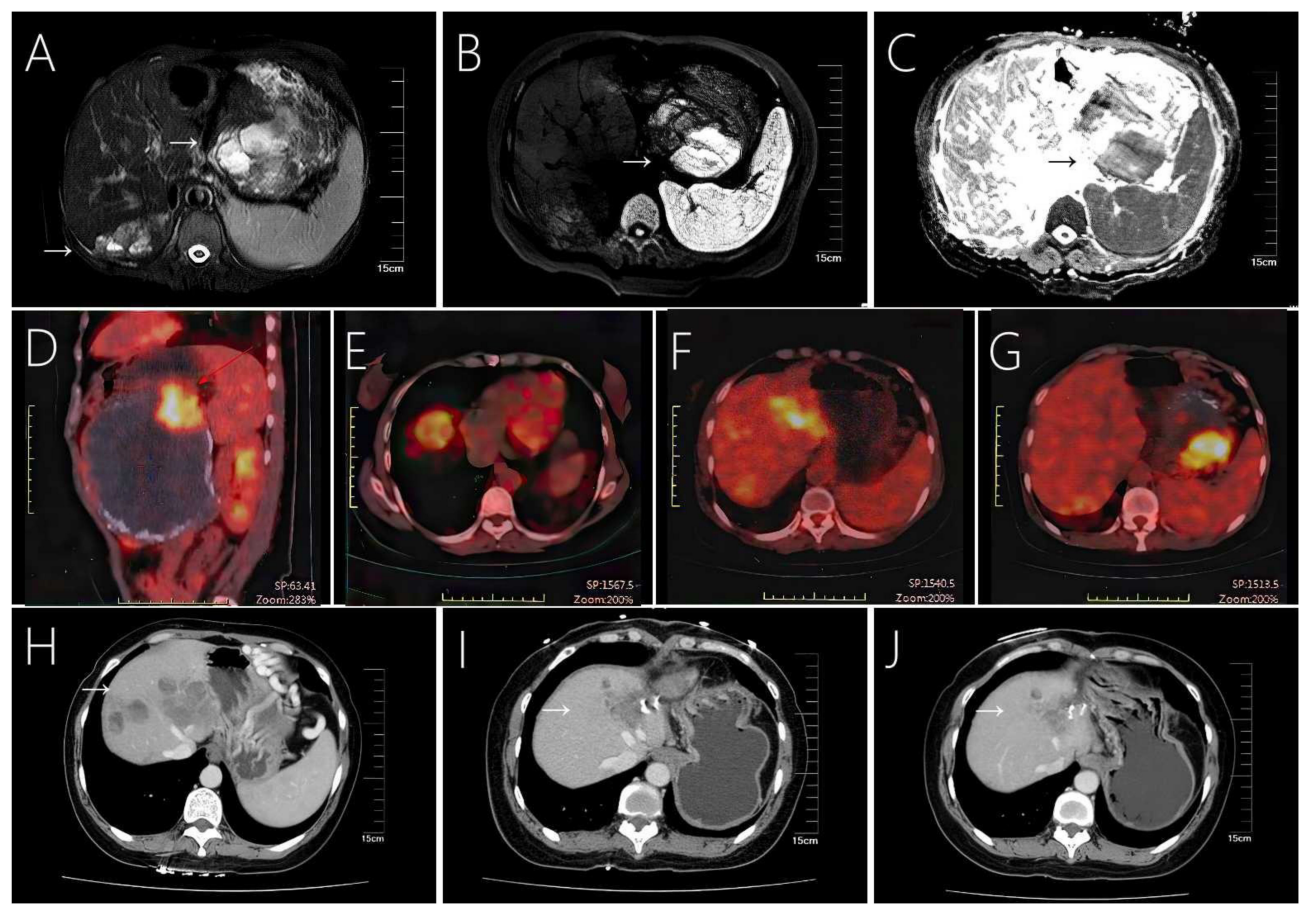
Case 8 shows a large solid pseudopapillary tumor in the tail of the pancreas with extensive multiple liver metastases, **(A)** showing a mixed cystic and solid structure. **(B, C)** MRI shows marked diffusion restriction within the primary tumor. **(D–G)** Tumors exhibit eggshell-like calcifications and increased radioactive uptake on PET-CT. Enhanced CT scan showed that the patient’s liver metastases existed in the same part of the tumor in the case of resection of the primary tumor in the patient **(H)** before surgery, **(I)** 20 months after surgery, and **(J)** 28 months after surgery, decrease or disappear.

The demographic characteristics of the eight patients enrolled in this study are shown in **Table 1**. The mean age (± standard deviation) of the patients was 32.4± 12.0 years (9 to 52 years). Clinical manifestations included a history of abdominal pain in five cases (62.5%), abdominal distention in one case (12.5%) and two (25%) asymptomatic cases. One patient (case 6) presented with acute symptoms of severe abdominal pain due to rupture of the primary tumor, but no clinical symptoms of recurrent liver metastases at 78 months after resection of the primary lesion. All patients underwent preoperative computed tomography (CT) or magnetic resonance imaging (MRI) scans (**Figures 1**–**4**), were surgically treated over a subsequent period of 3–12 days, and followed up postoperatively. The pancreatic lesions of all patients were in the body and tail of the pancreas. Tumor diameter ranged from 4.4 to 16.0 cm. Patients presented with a minimum of two foci of hepatic metastases, the diameter of which ranged from 2.0 to 5.6 cm. At the time of diagnosis, pancreatic tumors and hepatic metastases coexisted in six cases (75%), whereas in two patients (25%) hepatic metastases appeared, respectively, 22 and 78 months after resection of the primary tumor. In this patient series, seven cases (87.5%) presented resectable metastases and one case (12.5%) presented unresectable metastases in the liver. One patient (case8) showed shrinkage and disappearance of remaining liver metastases, after palliative tumor reduction surgery. Eight patients did not undergo anti-tumor treatment other than surgery.

**Table 1:** Clinical data of 8 patients with SPN with hepatic metastases.

Case No.	Age (y)/sex	Initial symptoms	pancreatic tumors		Hepatic metastases			Other metastasis	Preoperative pathological biopsy	Surgical approach	Extent of resection	Follow-up (months)
			Site	Size (cm)	Number	Diameter max. (cm)	Time					
1	31/F	Abdominal pain	body - tail	6.7	4	4.8	Initial diagnosis	None	Yes	distal pancreatectomy, splenectomy, partial hepatectomy	GTR	Alive,46
2	36/F	Abdominal pain	body - tail	10.2	2	2.5	Initial diagnosis	None	Yes	distal pancreatectomy, splenectomy, partial hepatectomy	GTR	Alive,97
3	23/F	Abdominal pain	body - tail	12.8	3	3.2	Initial diagnosis	None	Yes	distal pancreatectomy, splenectomy, partial hepatectomy	GTR	Alive,32
4	52/F	None	body - tail	4.4	3	2.0	Initial diagnosis	None	Yes	distal pancreatectomy, splenectomy, partial hepatectomy	GTR	Alive,67
5	30/F	Abdominal pain	body - tail	8.0	4	3.5	Initial diagnosis	None	Yes	distal pancreatectomy, splenectomy, partial hepatectomy	GTR	Alive,85
6	9/F	Abdominal pain and shock	body - tail	10.5	3	3.1	78 months post-operative	Colon and greater omentum	Yes	First time: distal pancreatectomy, splenectomy Second time: partial hepatectomy and Excision of lesions of the colon and greater omentum	GTR	Alive,138
7	36/F	None	body - tail	10.0	4	3.6	22 months post-operative	None	Yes	First time: distal pancreatectomy, splenectomy Second time: partial hepatectomy	GTR	Alive,65
8	42/F	Abdominal distention	body - tail	16.0	countless	5.6	Initial diagnosis	None	Yes	distal pancreatectomy, splenectomy, left lateral hepatectomy	STR	Alive,28

### 3.2 Preoperative diagnosis

Preoperative imaging diagnosis: We observed five cases of SPN (62.5%), one case of neuroendocrine tumor (12.5%), one case of mucinous cystic tumor (12.5%), and one case of pancreatic ductal adenocarcinoma (12.5%). Patients with hepatic metastases from SPN underwent preoperative ultrasound-guided liver tissue biopsy, and the results indicated that the liver tumors were consistent with hepatic metastases from SPN.

### 3.3 Surgical procedure and findings

**Figure 5:**
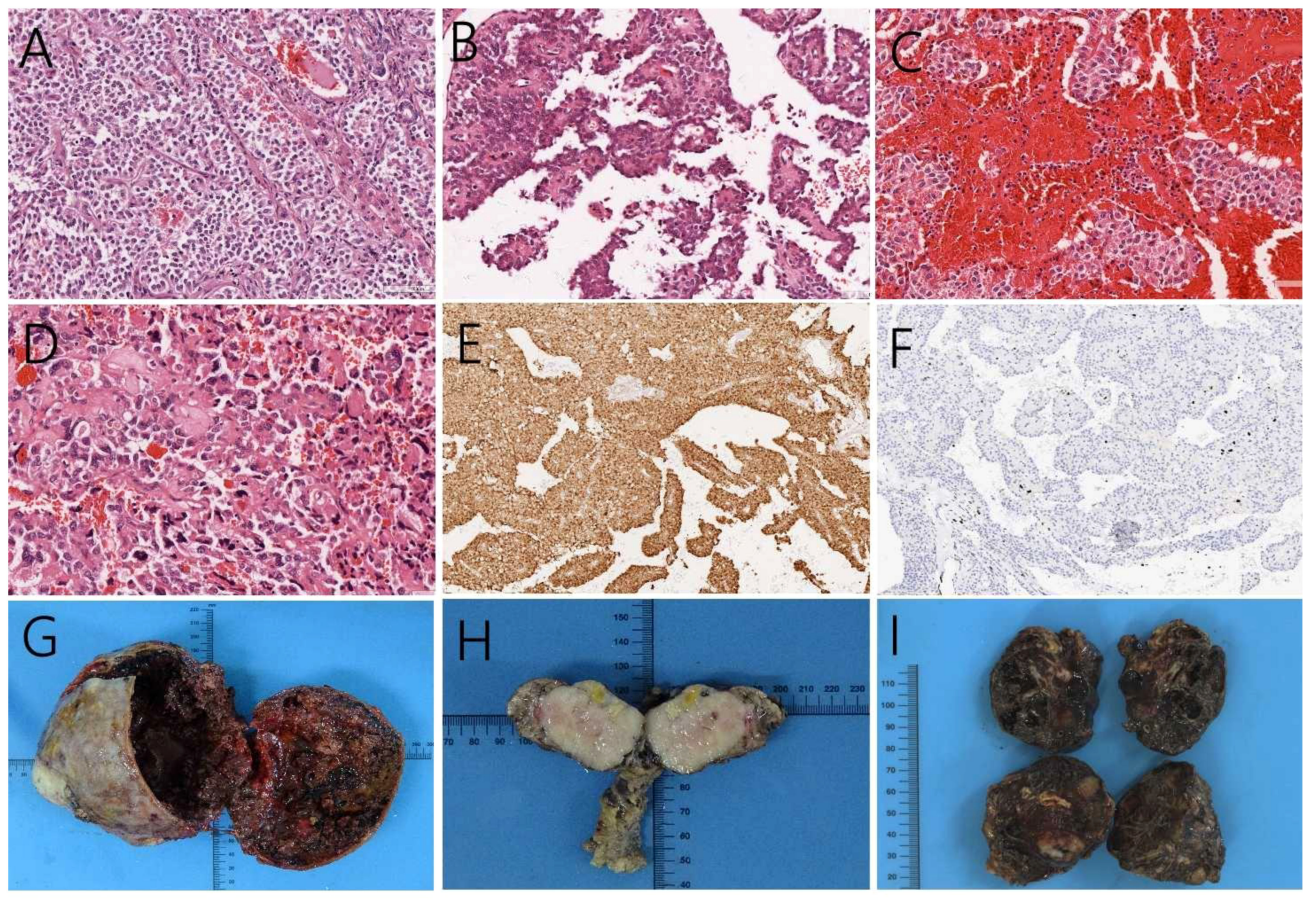
Positive hematoxylin-eosin staining, magnification 200×, **(A)** showing the tumor cells in the solid region. Cells were nested and presented similar morphology and size, without obvious atypia. **(B)** cells surrounding the small vessels form a pseudo-papillary neoplasm, as well as **(C)** hemorrhage and necrosis inside the tumors. **(D)** The cellular atypia of liver metastases had higher nuclear grade and more pronounced atypia than the primary tumor. **(E)** Immunohistochemistry, magnification 100×, showed that the tumor expressed nuclear β-catenin. **(F)** case 8 Ki-67 was observed in up to 5% of the tumor. **(G)** Larger pancreatic tumors showed mixed cystic and solid structures with a clear fibrous pseudo-envelope. Smaller tumors did not exhibit obvious cystic lesions, and the surrounding contours were less clearly defined. **(H)** Moreover, these tumors did not appear to be enveloped. **(I)** Hepatic metastases presented as brown, smooth-surfaced masses, locally grayish-white, with scattered cystic cavities on the cut surface.

All patients underwent open surgery, with complete resection of the lesion in seven cases, and palliative surgical resection in one case. Surgery included open exploration followed by resection of the primary tumor of the pancreas, in combination with splenectomy and enucleation/excision of hepatic metastases. Larger tumors usually present as well-defined disease borders, showing a round or round-like mass with a fibrous pseudo-envelope around the mass (**Figure 5G**). Smaller tumors did not exhibit obvious cystic lesions, and the surrounding contours were less clearly defined, Moreover, these tumors did not appear to be enveloped (**Figure 5H**). Hepatic metastases presented as masses with smooth surfaces, mostly brown in color. These further presented blood clotting-like structures, rigid, locally grayish-white, scattered cystic cavities on the cut surface (**Figure 5I**).

### 3.4 Histopathological findings

Histopathology revealed that in this case series pancreatic SPN cases consisted of an arrangement of solid and pseudopapillary regions. The tumor cells in the solid region were nested and in similar morphology and size, but without obvious atypia (**Figure 5A**). The tumors were rich in small blood vessels. Cells distant from the vessels appear to regress, whereas cells surrounding the small vessels form a pseudo-papillary arrangement around them (**Figure 5B**). We observed intratumoral areas of hemorrhage and necrosis (**Figure 5C**). For SPN with hepatic metastases, we found higher nuclear grade and more pronounced atypia than the primary tumor (**Figure 5D**). Furthermore, immunohistochemistry analysis showed nuclear expression of β-catenin (**Figure 5E**) and progesterone receptor, while estrogen receptor showed reduced expression. These confirm the diagnosis of SPN. The maximum value of Ki67 within this patient series was 5%, which was observed in case 8 (**Figure 5F**).

### 3.5 Follow-up

Postoperative follow-up was performed at least once a year for all eight patients (mean 70 months; range 28–138 months, **Table 1**), including routine clinical symptom and radiological assessments. The patient survival rate through the last follow-up in December 2021 was 100%. All patients showed the complete resolution of abdominal symptoms and no tumor recurrence or distant metastases. All patients reported improvements in the quality of life at the time of observation.

## 4 Discussion

The solid pseudopapillary neoplasm (SPN) of the pancreas is a rare, low-grade malignant indolent tumor that is most often observed in young women (mean age about 30 years) (9). Commonly, SPN tumors appear in the body and tail of the pancreas (10–12). Here we reported eight cases of hepatic metastases from solid pseudopapillary neoplasm of the pancreas that we reported. Similar to what is reported in the literature, our patient series was composed exclusively by women with a mean age of 32.4 years. Moreover, in agreement with previous reports, the primary tumors from patients in this series were located in the body and tail of the pancreas. Liver metastasis of SPN can occur synchronously or metachronously (13, 14). According to the literature, the rate of synchronous metastasis ranges between 0% and 4.3%, while the rate of metachronous metastasis ranges between 1.5% and 4.5% (14). We next discuss the problems and strategies for SPN and SPN with liver metastases.

### 4.1 Pathogenesis of SPN

Our series of patients one case (case 3) presented with a viral hepatitis C infection. Combined with previous studies, the pathogenesis of SPN may be associated with sex hormones (15), Wnt/β-catenin signaling pathways (1, 16), viral hepatitis (3). Hepatic metastases are commonly observed in several malignancies, including melanoma, lung, colorectal, pancreatic, and breast cancer. In contrast, hepatic metastases from SPNs of the pancreas are uncommon. For hepatic metastases, four key stages (microvascular, pre-angiogenic, angiogenic, and growth) have been identified during liver metastasis formation, where blood vessels are generated to provide oxygen and nutrient supply to the tumor cells. Interaction between tumor cells and tumor microenvironment plays an important role in tumor implantation, survival and metastasis (17). The higher survival rate and better prognosis of patients with SPN hepatic metastases, when compared to other malignancies, may be attributed to its indolent nature. However, the exact pathophysiological mechanisms need further investigation.

### 4.2 Clinical characteristics

Patients with SPN present with a wide range of symptoms at the time of diagnosis, with abdominal pain and distention being the most common symptoms. Strikingly, SPN patients rarely present evidence of pancreatic insufficiency, abnormal liver function or elevated tumor markers (1, 3, 18). However, abdominal pain is associated with malignant tumors. In fact, abdominal pain in SPN patients is an independent factor associated with malignancy (9). There are 5 cases in our case series that are supportive of this conclusion. Patients with advanced disease may present fatigue, weight loss, mild abdominal pain, or jaundice, depending on the burden of liver disease (17). A very rare cause of abdominal pain is the rupture of the tumor. In our series, patient 6 presented acute severe abdominal pain at the age of 9 years. The patient underwent emergency resection at a local hospital, with postoperative pathological observations suggestive of SPN. The patient was followed up regularly for the following 78 months (up to 15 years of age). During this period, the patient without abdominal symptoms presented with liver, colon, and large omental metastases, all of which were surgically removed. To the date of this study, the patient remained asymptomatic and showed no signs of recurrence. In patients presenting with a ruptured SPN, CT/MRI examination is essential to the correct diagnosis, which is confirmed by dissection and surgical findings (peritumoral hematoma or hematoma with disruption of tumor integrity). Most ruptured SPN originate from previous trauma. In contrast, spontaneous rupture is rare (**Table 2**). Among the cases presenting spontaneous rupture, two patients were in the gestational state and delivered full-term babies after surgery. In these cases, we hypothesized that progesterone receptor expression during pregnancy might have contributed to the rapid tumor growth (19, 23). The causes of spontaneous rupture are complex and may correlate with thickness of the capsule at the anterior part of the tumor, the size of the tumor and elevated intratumoral pressure. We observed that early detection and early intervention result in a good patient outcome. However, emergency surgery is not the first-line treatment given that SPN is a low-grade malignant tumor, thus emergency surgery may lead to a risk of recurrence in patients (case6). Therefore, in cases where conservative treatment can stabilize the patient’s condition, elective surgery may be a better option (20).

**Table 2:** Summary information on literature reported SPN spontaneous rupture.

Age (y)/sex	Abdominal trauma	Initial symptoms	Size(cm)	Treatment	Prognosis	References
21F (Pregnancy)	None	Abdominal pain and nausea	12	Distal pancreatectomy and splenectomy	Successful delivery 33 weeks after surgery	(19)
22F	None	Abdominal pain	8	Distal pancreatectomy and splenectomy	Normal recovery 10 days after surgery	(20)
13F	None	Severe abdominal pain	4	Conservative treatment and Surgery after 3 months	No recurrence after 2 years of follow-up	(21)
34F	None	Severe abdominal pain	12	Distal pancreatectomy	Died of organ failure 12 days after surgery	(22)
29F (Pregnancy)	None	Abdominal pain	17	Distal pancreatectomy and splenectomy	Successful delivery 8 months after surgery	(23)
31F	None	Abdominal pain and anemia	10	Distal pancreatectomy	No recurrence after 3 years of follow-up	(24)
9/F	None	Abdominal pain	10.5	Distal pancreatectomy and splenectomy	Recurrent liver, omentum, and colon metastases 78 months (15years old) after surgery	Case 6 of our patient series

### 4.3 Radiological presentation

Imaging tools such as ultrasound, CT and MRI scans are commonly used in the diagnosis of SPN and hepatic metastases. These tumors commonly appear as solid, cystic, or heterogeneous density masses, and liver metastases tend to be more specific than pancreatic primary tumors (**Figures 1A**, **2A**, **3A**, **4A**). Metachronous liver metastases can be divided into three types based on imaging: classic, inert, and invasive. Detection of those types can be challenging, and enhanced MRI has relatively high sensitivity (14). Despite advances in diagnostic methods, the preoperative diagnosis of SPN remains a clinical challenge. CT scans are used to determine the size and morphology of the mass, the pancreatic anatomy, as well as to ascertain the presence of local invasion and metastases (1, 3). In our series, we observed intratumoral or peritumoral eggshell-like calcifications, in four cases (**Figures 2A**, **4D**). The presence of peritumoral and hemorrhagic foci in the tumor mass helped distinguish it from other pancreatic tumors (25). MRI imaging is a preferred technique in diagnosing pancreatic lesions given that it provides further information about tumor tissue bleeding and necrosis (3, 6). In contrast, CT scan imaging cannot resolve papillary and cystic lesions, which are visible when using MRI.

### 4.4 Differential diagnosis

Previous reports describe a broad differential diagnosis of SPN, including neuroendocrine tumors, mucinous cystic neoplasms, pancreatic ductal adenocarcinoma, intraductal papillary mucinous neoplasm, lkymphangioleiomyoma, sarcomas, islet cell tumors, adenoid cell cystic adenocarcinoma and pseudocysts (1, 26–28). Since the differential diagnosis of these primary tumors is not the focus of our attention, this will not be discussed in depth in this review. However, it is necessary to identify and diagnose hepatic metastases before surgery. For patients with hepatic metastases from pancreatic tumors, lung or breast cancer, resection of the primary site may be inappropriate even in the case of small hepatic metastases, regardless of the number and location of liver metastases (17). Therefore, we performed preoperative ultrasound-guided liver tissue biopsy in all hepatic metastases, and observed those were consistent with SPN. Although some studies suggest a puncture biopsy of the primary tumor, we did not perform it given the risk of severe acute pancreatitis, due to possible damage to the pancreatic duct (29). In contrast, liver metastases biopsy imposes fewer risks for the patients. Preoperative percutaneous aspiration biopsy and endoscopic ultrasound fine-needle aspiration (EUS-FNA) in establishing an accurate preoperative diagnosis may also lead to tumor cell dissemination (30). All eight biopsies in this group showed no tumor spread or complications.

### 4.5 Histopathological characteristics

SPN is composed of an arrangement of solid and pseudopapillary areas, presenting hemorrhage and necrosis within the tumor. Positive immunohistochemistry for β-catenin (nuclear)and progesterone receptor are very helpful for the diagnosis of SPN (1). A recent study found that frequent and diffuse expression of lymphoid enhancer factor 1 (LEF1) in the nucleus is associated with SPN (31). Therefore, we will expand the characterization of our series of tumors by studying LEF1 involvement in tumor development. When detecting hepatic metastases, hematoxylin-eosin staining of metastases with typical histological features similar to those of the primary lesion allows for accurate diagnosis (17). Immunohistochemical staining is more helpful in differentiating SPN from the more common tumors such as pancreatic acinar cell carcinoma and neuroendocrine tumors (6).

### 4.6 “Malignant” SPN

The definition and the prediction of SPN malignancy are still challenging (32). In the clinical setting, SPN should be defined as malignant only on the basis of recurrence after surgical resection (12). Therefore, many studies have focused on potential predictors of recurrence, including age <13.5 years (33), male gender (34), high neutrophil/lymphocyte ratio (35), large tumor size (4, 36, 37), positive surgical margins (33), Ki-67≥4% (38, 39), lymphovascular invasion (37), malignant presentation in imaging analysis (40) and malignant features in pathological observations (4). In contrast, several studies have reported that no clinicopathological factors may predict tumor behavior (12, 41, 42). Conversely, in the pathological context, indicators of tumor malignancy include lymph node or distant metastases, cellular anisotropy, envelope invasion, parenchymal invasion, neurological or lymphovascular invasion, and extra-pancreatic invasion (10, 18). These indicators provide a potential clue to tumor recurrence. Moreover, pathologically benign indicators may also recur several years after surgical resection. Therefore, the WHO classifies all SPN as low-grade malignant tumors, regardless of microscopic malignant features (43). Our team also projected the population that would be at high risk for SPN. In multivariate analysis, clinical symptoms, unclear tumor margins, incomplete tumor capsules, maximum tumor diameters of ≥ 7.2 cm, and prognostic nutritional index values of < 47.45 were independent risk factors for SPNs in high-risk groups. A nomogram model was successfully established to predict groups at high risk of SPNs (44).

### 4.7 Surgery

Surgery remains the most effective treatment option for SPN hepatic metastases (9). Complete resection of hepatic metastases is possible in cases with limited liver involvement (12). The onset of liver metastases varies with patients, ranging from a minimum of 2 months (45) to a maximum of 168 months (46). In our series, liver metastases coexisted with the primary tumor in six patients at the time of presentation, and two patients showed recurrence of liver metastases, after resection of the primary lesion, at 22 and 78 months, respectively. For resectable hepatic metastases, negative surgical margins are the primary treatment, associated with patient survival over 5 years, similar to that of patients without metastases admitted to surgery (1, 6, 47). In patients with unresectable hepatic metastases, surgical resection of primary and metastatic lesions to alleviate patients’ clinical symptoms and tumor burden, together with postoperative multimodality therapy for hepatic metastases to control liver tumor size and intra- and extrahepatic metastases may be an efficient approach (6, 48). If the growth pattern of the liver metastases is inert, the treatment options may include observation or radiofrequency ablation (14).

### 4.8 Chemotherapy

Despite the reports showing promising results in SPN patients submitted to chemotherapy, a truly efficient chemotherapeutic regimen is still to be developed (1, 49). 5-Fluorouracil (5FU) and gemcitabine are the most commonly used chemotherapy drugs (18). Strauss et al. reported the use of 6 cycles of neoadjuvant chemotherapy with cisplatin and 5FU to induce tumor shrink and disappearance, as well as to enable surgical removal of otherwise unresectable SPN (50). Maffuz et al. obtained similar results with seven cycles of gemcitabine combined with radiation therapy (51). Still, several reports suggest that chemotherapy may indeed lead to uncontrolled tumor growth (4, 52). Previous studies reported data on the use of targeted drugs, including an mTOR inhibitor (everolimus) (53) and a multi-targeted receptor tyrosine kinase inhibitor (sunitinib) (54).

### 4.9 Radiotherapy

A small number of reports suggest that radiation therapy is beneficial for SPN patients with incomplete resection (1). Zauls et al. described a 33-year-old patient with unresectable SPN, treated with radiotherapy. Intermittent tumor reduction was observed using abdominal MRI during follow-up every 6 months (55). Moreover, two case reports demonstrated radiosensitivity by controlling the progression of SPN with radiation therapy (56, 57). However, radiotherapy used for unresectable tumors or as adjuvant therapy after tumor removal is not common in clinical practice (40).

### 4.10 Other treatments

Radiofrequency ablation (6), hepatic artery embolization (58), targeted therapy (54), liver transplantation (59), chemosaturation therapy with percutaneous hepatic perfusions (60), and anti-estrogenic drugs (27) have been successfully used in pancreatic SPN treatment with liver metastases. For hepatic metastases, systemic therapy regimens, new biological therapies, and immunotherapy have revolutionized patient prognosis. In the future, the combination of these with genetic testing might provide more accurate information to guide clinical decisions and predict the prognosis of patients with hepatic metastases (17).

### 4.11 Prognosis

In our study, patients with SPN presented good prognosis, (median follow-up of 70 months) with 6 out of 8 cases followed for more than 4 years. During the follow-up period we observed no further tumor recurrence or distant metastasis in repeated abdominal enhancement CT. All patients showed complete reduction in abdominal symptoms after surgery. Nevertheless, SPN is a low-grade malignant tumor, thus being at risk of recurrence or the generation of metastasis, despite being completely resected. The average tumor recurrence time has been reported to be above 4 years after resection (18). Thus, we propose that the follow-up period for SPN patients should be a minimum of 5 years after primary tumor resection. Failure to follow-up over time may result in inadequate treatment. One of the patients included in our study (case 8) showed heterogeneous liver metastases shrinkage and disappearance, after resection of the primary pancreatic tumor. The patient further showed metastases reduction in the spleen and the left outer lobe of the liver without additional treatment of other hepatic metastases during 20 months of follow-up (**Figures 4H–J**). Spontaneous regression of SPN occurs rarely (published data reviewed and summarized in **Table 3**). Spontaneous tumor regression has been observed in patients between 9 and 18 years of age (61–64). We hypothesize that during this age period, the liver outgrows the tumor, due to its metabolic activity, which is significantly higher than that of a slow-growing tumor such as SPN, likely inducing persistent degenerative changes in the tumor (such as hemorrhage, necrosis, and resorption). In this patient series, case 8 (42-year-old woman) shows regression of hepatic metastases after resection of the primary tumor. The reason may be attributed to host factors, which are considered to be associated with the immune system. Removal of the primary tumor may favor the balance towards the host, prompting the immune system to control residual disease. In conclusion, we propose that spontaneous regression of SPN might have multiple underlying mechanisms, and that additional research is necessary to deepen our knowledge in this patient outcome. In patients with liver metastases, the chance of long-term survival appears to be greater when the metastatic tumor is treated with resection. The prognosis of treated SPN with liver metastases usually exceeds 5 years (ranging from 6 months to more than 17 years) (1, 65). Despite metastases, surgical tumor reduction can achieve a satisfactory outcome. Because of the relatively inert behavior of these tumors, even in locally advanced or metastatic disease, or after the re-excision of recurrent disease, lifelong follow-up must be performed annually if the patient is suitable for surgery (65).

**Table 3:** Summary information on literature reported SPN spontaneous regression.

Age (y)/sex	Initial symptoms	Hepatic metastases	Size(cm)	Treatment	Follow-up(months) And Tumor changes	References
15F	Severe epigastric pain	None	4	None	18 Tumor disappearance	(61)
9F	Nausea and abdominal pain	None	4.2	None	12 Tumor disappearance	(61)
13M	Abdominal pain	None	5.1	None	72 Tumors cannot be measured	(62)
18F	Abdominal pain	None	4.5	None	120 Tumor shrinks from 4.5cm to 1.5cm	(63)
14F	epigastric pain	Three masses in the right lobe (maximum diameter: 6.0 cm)	11	None	156 Pancreatic tumor decrease and hepatic metastases disappear	(64)
42F	Abdominal distention	Mostly in the left and right lobes (maximum diameter: 5.6 cm)	16.0	Palliative tumor reduction surgery	28 Reduction and. disappearance of hepatic metastases	Case 8 of our patient series

## 5 Conclusion

Hepatic metastases from solid pseudopapillary neoplasms of the pancreas are rare, and for hepatic metastases, preoperative biopsy is required when the diagnosis is uncertain. Total surgical resection is the most effective treatment for resectable hepatic metastases. In patients with unresectable hepatic metastases, the survival benefit achieved by aggressive tumor reduction surgery is encouraging and potentially results in long-term survival. Given the lack of reports of hepatic metastases from pancreatic solid pseudopapillary neoplasms, additional case reports and long-term follow-up studies are needed to fully understand the pathogenesis of these tumors and their metastases. This paper presents clinical experiences with SPN-related hepatic metastases at the Affiliated Hospital of Jilin University, which can be used to guide patient counseling in clinical practice. At the same time, our study can serve as an additional reference to the few available cases as a prospective guide for clinicians.

## Author contributions

XL: Conceptualization, Data curation, Writing – original draft. JR: Supervision, Writing – original draft. JK: Writing – original draft. PJ: Formal analysis, Writing – original draft. LG: Formal analysis, Investigation, Writing – review & editing. LZ: Methodology, Software, Writing – review & editing. WH: Project administration, Investigation, Writing – review & editing. YL: Project administration, Visualization, Writing – original draft. BJ: Data curation, Project administration, Resources, Writing – original draft.
